# Integrated analysis of DNA methylome and transcriptome revealing epigenetic regulation of *CRIR1*-promoted cold tolerance

**DOI:** 10.1186/s12870-024-05285-0

**Published:** 2024-07-05

**Authors:** Zhibo Li, Wenjuan Wang, Xiaoling Yu, Pingjuan Zhao, Wenbin Li, Xiuchun Zhang, Ming Peng, Shuxia Li, Mengbin Ruan

**Affiliations:** 1 National Key Laboratory for Tropical Crop Breeding, Key Laboratory of Biology and Genetic Resources of Tropical Crops, Institute of Tropical Bioscience and Biotechnology, Sanya Research Institute of Chinese Academy of Tropical Agricultural Sciences, Haikou, 571101 P.R. China; 2 College of Tropical Crops, Hainan University, Haikou, 570228 P.R. China

**Keywords:** RNA-seq, Cold stress, Cassava, *CRIR1*, DNA methylation

## Abstract

**Background:**

DNA methylation contributes to the epigenetic regulation of nuclear gene expression, and is associated with plant growth, development, and stress responses. Compelling evidence has emerged that long non-coding RNA (lncRNA) regulates DNA methylation. Previous genetic and physiological evidence indicates that lncRNA*-CRIR1* plays a positive role in the responses of cassava plants to cold stress. However, it is unclear whether global DNA methylation changes with *CRIR1*-promoted cold tolerance.

**Results:**

In this study, a comprehensive comparative analysis of DNA methylation and transcriptome profiles was performed to reveal the gene expression and epigenetic dynamics after *CRIR1* overexpression. Compared with the wild-type plants, *CRIR1*-overexpressing plants present gained DNA methylation in over 37,000 genomic regions and lost DNA methylation in about 16,000 genomic regions, indicating a global decrease in DNA methylation after *CRIR1* overexpression. Declining DNA methylation is not correlated with decreased/increased expression of the DNA methylase/demethylase genes, but is associated with increased transcripts of a few transcription factors, chlorophyll metabolism and photosynthesis-related genes, which could contribute to the *CRIR1*-promoted cold tolerance.

**Conclusions:**

In summary, a first set of transcriptome and epigenome data was integrated in this study to reveal the gene expression and epigenetic dynamics after *CRIR1* overexpression, with the identification of several TFs, chlorophyll metabolism and photosynthesis-related genes that may be involved in *CRIR1*-promoted cold tolerance. Therefore, our study has provided valuable data for the systematic study of molecular insights for plant cold stress response.

**Supplementary Information:**

The online version contains supplementary material available at 10.1186/s12870-024-05285-0.

## Introduction

DNA methylation at the C5 position of cytosine (5-methylcytosine, 5mC) is conserved in plants and mammals, and contributes to the epigenetic regulation of nuclear gene expression and the modulation of chromatin structure [1, 2]. It has been observed that DNA methylation is associated with many biological processes, including embryonic development, fruit ripening, and environmental stress response. Disrupted DNA methylation can lead to developmental abnormalities among mammals and plants [1]. In plants, 5mC can appear not only in the symmetric CG and CHG sequence contexts but also in the asymmetric CHH sequence contexts (in which H represents A, T, or C). Two distinct pathways, namely, the de novo methylation pathway and the maintenance methylation pathway, are involved in establishing and maintaining the marks of DNA methylation [1]. In *Arabidopsis*, the symmetric CG-site methylation is maintained by the METHYLTRANSFERASE 1 (MET1) during DNA replication, which recognizes hemi-methylated CG dinucleotides and induces unmodified cytosine methylation following DNA replication [3, 4]. CHROMOMETHYLASE 2 and 3 (CMT2 and CMT3) play a leading role in catalyzing CHG methylation [5, 6]. CHH methylation is performed by CMT2 or domain-rearranged methyltransferase (DRM1 and DRM2) depending on the genomic region. DRM2 maintains CHH methylation through a plant-specific mechanism, RNA-directed DNA methylation (RdDM) pathway, in which 24 nt small interfering RNAs (siRNAs) are required [2, 6, 7]. Specific DNA methylation states are dynamically regulated by DNA methylation and demethylation reactions. Active DNA demethylation requires 5’-methylcytosine DNA glycosylase/lyase enzymes, including REPRESSOR OF SILENCING 1 (ROS1), DEMETER (DME), DEMETER-LIKE 2 (DML2), and DML3, which can erase DNA methylation through a base excision repair pathway [8, 9].

Most plants with sessile structures cannot avoid external stresses from the natural environment during their entire life cycles. Freezing and chilling conditions or extremely low temperatures are key factors affecting plant productivity and quality, directly inhibiting enzyme activities and damaging cell membranes and eventually leading to cell death. Under these circumstances, plants have evolved various systems at the molecular, cellular, and physiological levels to deal with cold stress [10]. Many molecular changes under cold stress, including transcriptional and metabolic changes that increase or decrease levels of specific RNAs, proteins, phytohormones, and metabolites, have been observed. Down-regulated genes include photosynthesis-related genes and growth regulators [11]. However, up-regulated genes encoding protein kinases, transcription factors and enzymes, which are involved in signal transduction and metabolites synthesis in cold stress responses and further regulation of gene expressions [10]. For example, APETALA2/ethylene-responsive factor (AP2/ERF) transcription factors, such as Dehydration-response element-binding proteins (DREBs), that bind to DRE element and act as master regulators in cold-inducible gene expression [12]. In addition to the *DREB* signaling pathway, other pathways, such as Heat-shock transcription factor A1 (HSFA1) can interacts with NONEXPRESSER OF PR GENES 1 (NPR1), which is a key component of the SA signaling pathway, to promote cold acclimation by activating the expressions of target genes under cold stress [13].

Cassava (*Manihot esculenta* Crantz) is recognized as one of the essential crops with starchy roots as a major source of carbohydrates for more than 800 million people worldwide. This crop has been widely cultivated in the tropics of South America, Africa, and Asia with its high yield potential and growth capacity in poor soils [14–16]. So far, root starch of cassava has not only been used in human food, but also in the pharmaceutical, paper, textile, and biofuel industries [16, 17]. Since 2000, there have been strong demands for cassava in each year, however, its potential yields are limited by susceptibility to abiotic and biotic stresses [16]. In particular, cold stress lead to severe yield losses [18]. Previous studies have shown the influences of cold stress on the normal development of cassava plants [19]. To illustrate, cold stress can increase proline, malondialdehyde (MDA), soluble sugars, and ROS levels, and negatively constrain chlorophyll contents [20, 21]. Recent studies show that a number of cassava protein-coding and non-coding RNAs involved in multiple processes play an important role in the adaptation of plants to cold stress. For example, *MeCBF1* overexpression confers tolerance to cold stress in transgenic *Arabidopsis* and cassava plants [22, 23]. RNAi-driven repression of MeMYB2 results in low-temperature tolerance of transgenic cassava plants [24]. Previously, we identified a cold-induced long non-coding RNA, *CRIR1*, which can enhance the cold tolerance of transgenic cassava plants. Transcriptome analysis demonstrated that CRIR1 regulates a range of cold stress-related genes in a CBF‐independent pathway [25]. However, how *CRIR1* affects the expression of these genes remains unknown. Whether the genome-wide DNA methylation dynamics are involved in *CRIR1*-induced gene expression alteration has not been characterized.

In this study, a whole-genome bisulfite sequencing analysis was performed on wild-type (WT) plants and *CRIR1*-overexpressing (OE) plants, with an interesting alteration of DNA methylation revealed. Further analysis suggests that *CRIR1* does not mediate DNA hypo-/hypermethylation at a subset of genes by regulating the expression of methylase or demethylase genes. By comparing the transcriptomes and DNA methylomes, we found that decreased gene body methylation in *CRIR1*-overexpressing plants is associated with hyperactivation of these genes, which may also responsible for cold tolerance of the transgenic plants. This study has provided a new insight and a molecular foundation for understanding the underlying mechanisms of *CRIR1*-induced cold resistance in plants.

## Results

### DNA methylome features of *CRIR1*-overexpressing plants

To investigate whether the genome-wide DNA methylation dynamics are involved in *CRIR1*-induced gene expression alteration, a whole-genome bisulfite sequencing was performed on the shoot buds and young leaves collected from transgenic plants and WT plants. For each sample, at least 200 M paired-end reads (read length = 150 bp) were generated. Approximately 75% of clean reads were mapped to the reference genome using BSMAP, covering more than 98% of the genome. Importantly, the results show that bisulfite conversion rates are over 99.2% in all samples (Table 1). All of sequenced methylomes present an average coverage of around 30 folds per DNA strand, indicating that those methylome data obtained were reliable and accurate. Approximately 92% of cytosines were covered by at least five reads and more than 80% of genome was covered by at least 30 reads.

**Table 1:** Summary of whole-genome bisulfite sequencing data

Sample	Clean reads	Mapped Ratio(%)	Number of methylated cytosines			Conversion rates(%)
			mCG	mCHG	mCHH	
WT	165,372,532	75.67	7,355,933	9,845,219	7,390,649	99.27
#1	201,303,078	75.38	7,068,016	9,473,184	7,529,117	99.28
#5	207,692,626	73.98	7,153,737	9,661,019	7,726,702	99.30

**Fig. 1:**
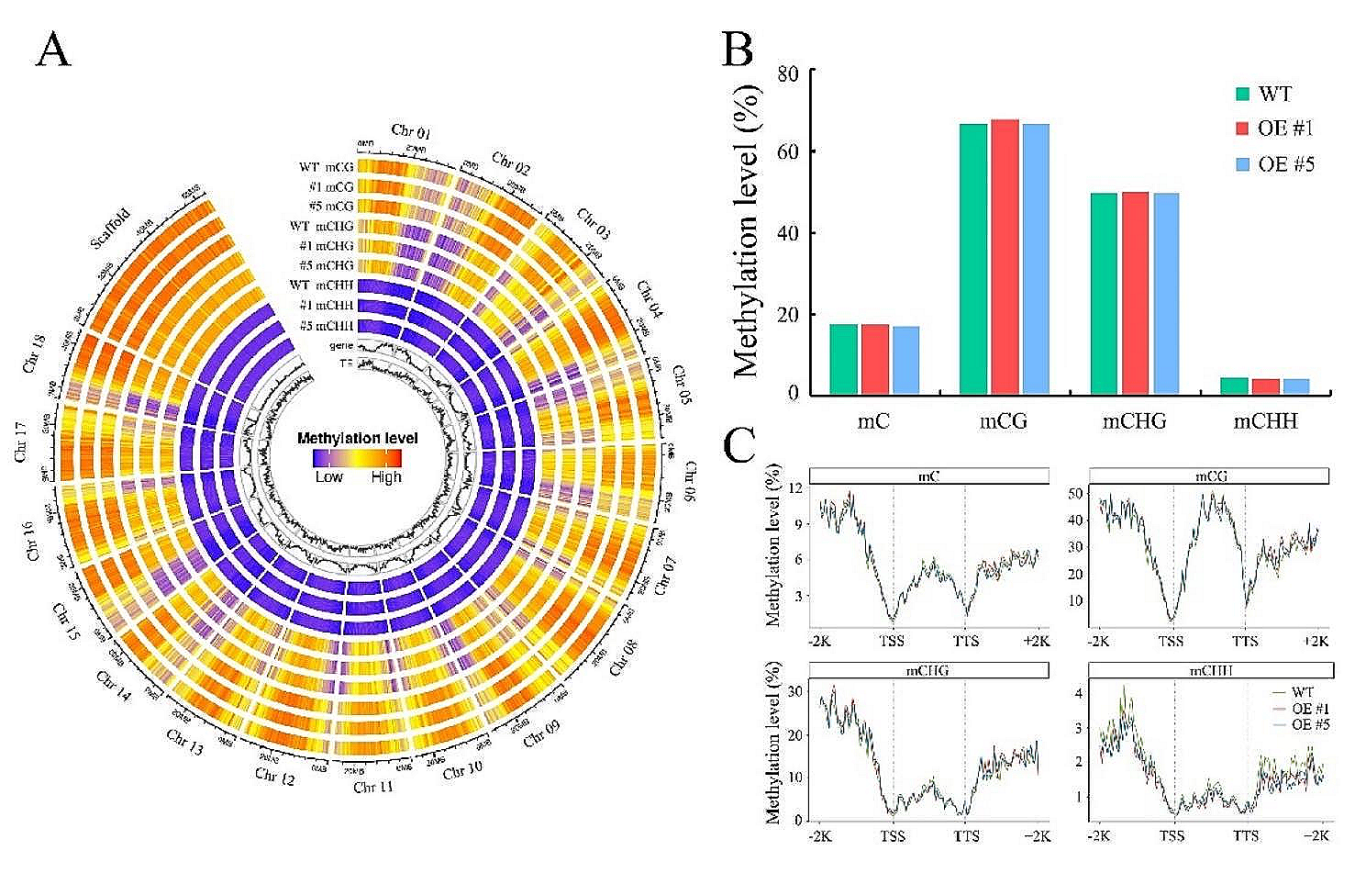
Genome-wide DNA methylation profiles of wild-type and *CRIR1*-overexpressing cassava plants. **(A)** Circle plots of DNA methylation levels in CG, CHG, CHH contexts across 18 chromosomes of WT and *CRIR1*-overexpressing #1 and #5 lines. Red color indicates a high methylation level, and blue color indicates a low methylation level. **(B)** Average methylation levels of *CRIR1*-overexpressing (OE) #1, #5 and WT in all three contexts. **(C)** Average levels of mC, mCG, mCHG, and mCHH methylation in all genes of OE #1, OE #5 and WT, including 2 kb upstream of transcription start site (TSS) and 2 kb downstream of transcription termination site (TTS)

Global DNA methylation profiles of chromosomes 1 to 18 are shown in Fig. 1A. Average DNA methylation levels of each sample were calculated. It shows that the average genome-wide methylation levels of CG, CHG, and CHH are 66.7%, 49.5%, and 4.2%, respectively (Fig. 1B). Among them, the CG context presents the highest methylation level, which is consistent with the methylation proportion previously discussed [26, 27]. The DNA methylation levels of transgenic lines and WT are close to each other. Moreover, in order to explore the relationship between DNA methylation and gene expression, a comparison of the methylation levels in the gene body and flanking region was conducted. As shown in Fig. 1C, similar to other plant species [28, 29], these plant samples present sharply-dropping methylation levels around the transcription start site (TSS) and the transcription termination site (TTS) in those three sequence contexts. Significantly, CG context exhibits the highest methylation levels across the gene body and flanking regions, while CHG and CHH contexts exhibit higher methylation levels in the flanking regions than those in the gene body (Fig. 1C). Further analysis shows that there is no significance difference of methylation level in gene body, while CHH methylation levels at promoter and 2 kb flanking sequences of genes were reduced in *CRIR1*-overexpressing plants relative to WT (Fig. 1C).

### Identified differentially methylated regions (DMRs) in transgenic plants

**Fig. 2:**
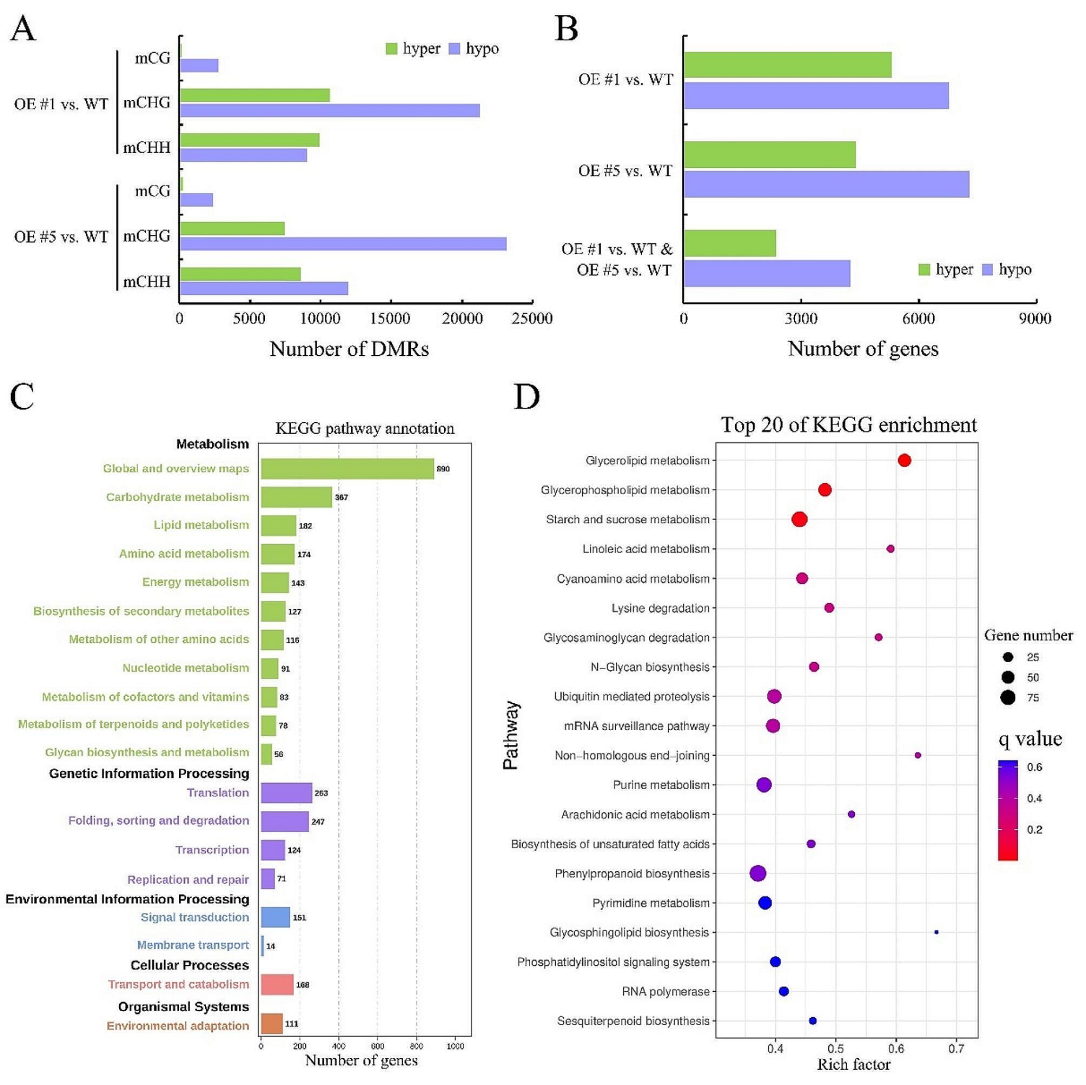
Identification of DMRs, DMR-related genes (DMGs) and their corresponding signaling pathways. **(A)** Relative numbers of hyper- and hypo-DMRs in the CG, CHG, and CHH contexts of OE lines relative to WT. **(B)** Numbers of DMGS between each pair of comparisons. **(C)** KEGG analysis of hypo-DMGs in *CRIR1* transgenic lines under. **(D)** Top 20 enriched KEGG pathways

To explore the dynamics of DNA methylation at specific locations in detail, we analyzed the differentially methylated regions (DMRs) among WT and transgenic plant lines. A total of 53,831 and 53,890 *CRIR1*-responsive DMRs (*p* < 0.05) were identified in OE #1 and OE #5 lines, respectively. All DMRs were classified into three contexts, with CHG and CHH contexts containing the majority of DMRs (Fig. 2A, Table S2). Further examination of methylation levels in three contexts revealed that hypermethylation mainly appears in CHG and CHH contexts, while hypomethylation appears in all contexts. In comparison to WT plants, transgenic plant lines presented much higher numbers of hypomethylated DMRs (hypo-DMRs) (Fig. 2A). Further analysis of hypo-DMR distribution among genes, TEs, and their flanking sequences, showed that hypo-DMRs are preferentially located in the gene body regions in CG and CHG contexts, but are particularly enriched in flanking regions of genes in the CHH context (Fig. S1).

Furthermore, genes with functional overlap of at least 1 bp with DMRs were selected and defined as DMR-related genes (DMGs) in this study (Fig. 2B). A total of 2,348 and 4,255 hyper-DMGs and hypo-DMGs were identified in both OE #1 and OE #5 lines, respectively. KEGG analysis revealed that hypo-DMGs in *CRIR1* transgenic lines are involved in metabolic processes, such as carbohydrate, lipid metabolism pathway, genetic information processing pathway, such as protein translation, folding, sorting and degradation pathway, environmental information processing, and cellular processes are also enriched, indicating a global response of metabolites, protein, and gene expression changes in transgenic plants (Fig. 2C). Those top 20 enriched pathways including glycerolipid metabolism, linoleic acid metabolism, and mRNA surveillance pathways, are shown in Fig. 2D.

### *CRIR1* does not mediate DNA methylation by regulating the expression of methylase or demethylase genes

**Fig. 3:**
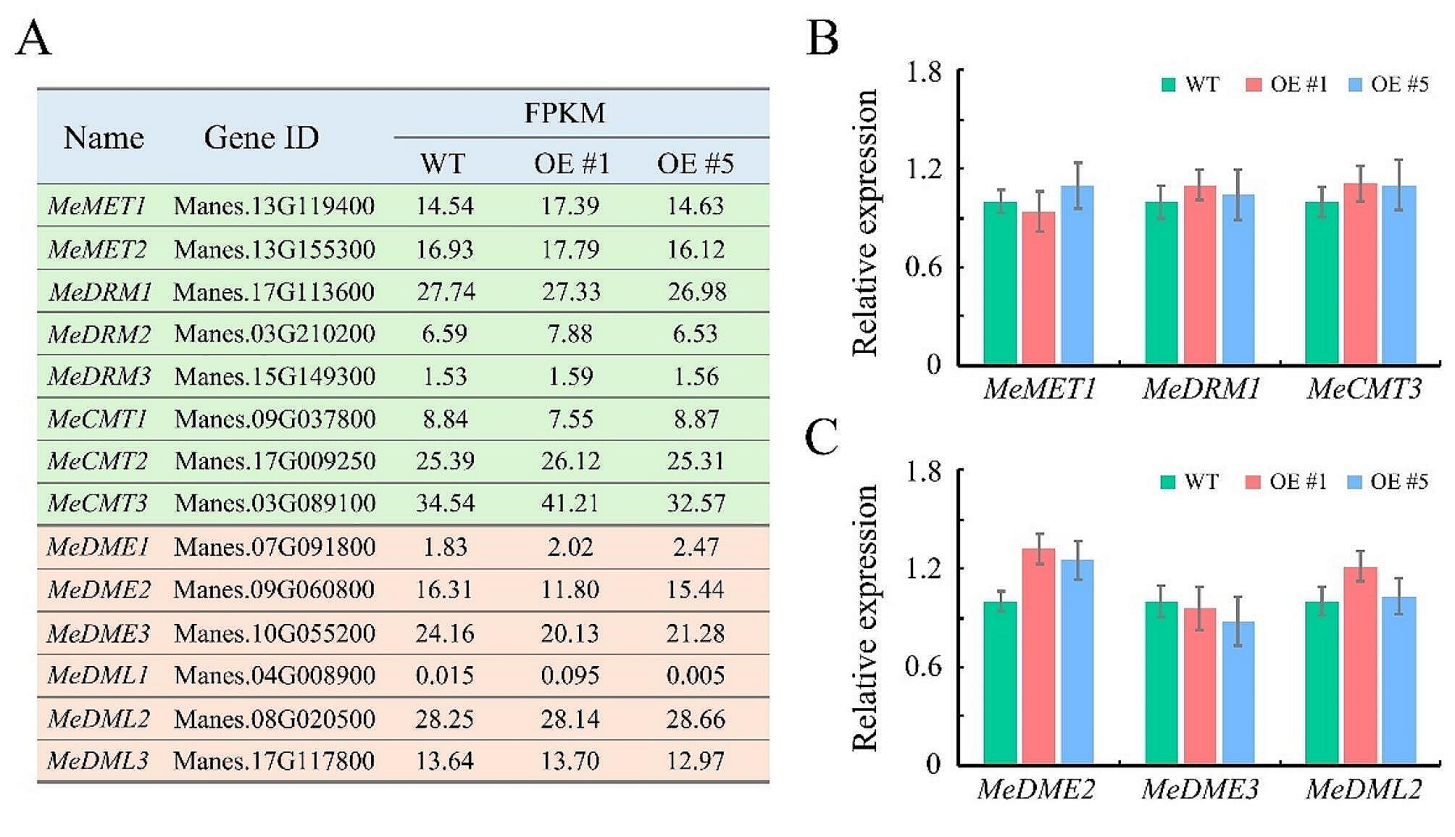
Expression of DNA methylation/demethylation related genes in WT and transgenic plants. **(A)** List of DNA methylation (green), and demethylation (pink) related genes and their corresponding FPKM values in RNA-seq data. **(B)** Expression levels of DNA methylation genes, including *MeMET1*, *MeDRM1* and *MeCMT3*, examined with qRT-PCR. **(C)** Expression levels of DNA demethylation genes, including *MeDME2*, *MeDME3* and *MeDML2*, examined with qRT-PCR. Data are provided as means ± SD of three biological replicates

DNA methylation is dynamically affected by activities of DNA methyltransferase and DNA demethylase [30]. A large number of hypo-DMRs which could result from enhanced DNA demethylase activities or decreased DNA methyltransferase activities, were identified in *CRIR1*-overexpressing plants. To investigate these possible reasons, we examined the expression levels of DNA methyltransferase and demethylase genes in transgenic plants. Eight genes of methyltransferase orthologs in cassava genome, including *MeMET1/2*, *MeDRM1/2/3*, and *MeCMT1/2/3*, were identified in this study. Among these eight genes, none has significantly increased or decreased transcript levels in transgenic plants, neither in RNA-seq nor in qRT-PCR data (Fig. 3A-B). In addition, six cassava DNA demethylases, including *MeDME1/2/3* and *MeDML1/2/3*, were identified. Among these demethylases, *MeDML1* is barely expressed (fragments per kilobase of transcript per million mapped reads, FPKM < 1) in shoots and young leaves of cassava, while the others are neither induced nor repressed by *CRIR1* (Fig. 3A, C). These results indicate that *CRIR1* does not mediate DNA methylation by regulating the expression of methylase or demethylase genes.

### *CRIR1*-hyperactivated genes are correlated with their decreased DNA methylation

**Fig. 4:**
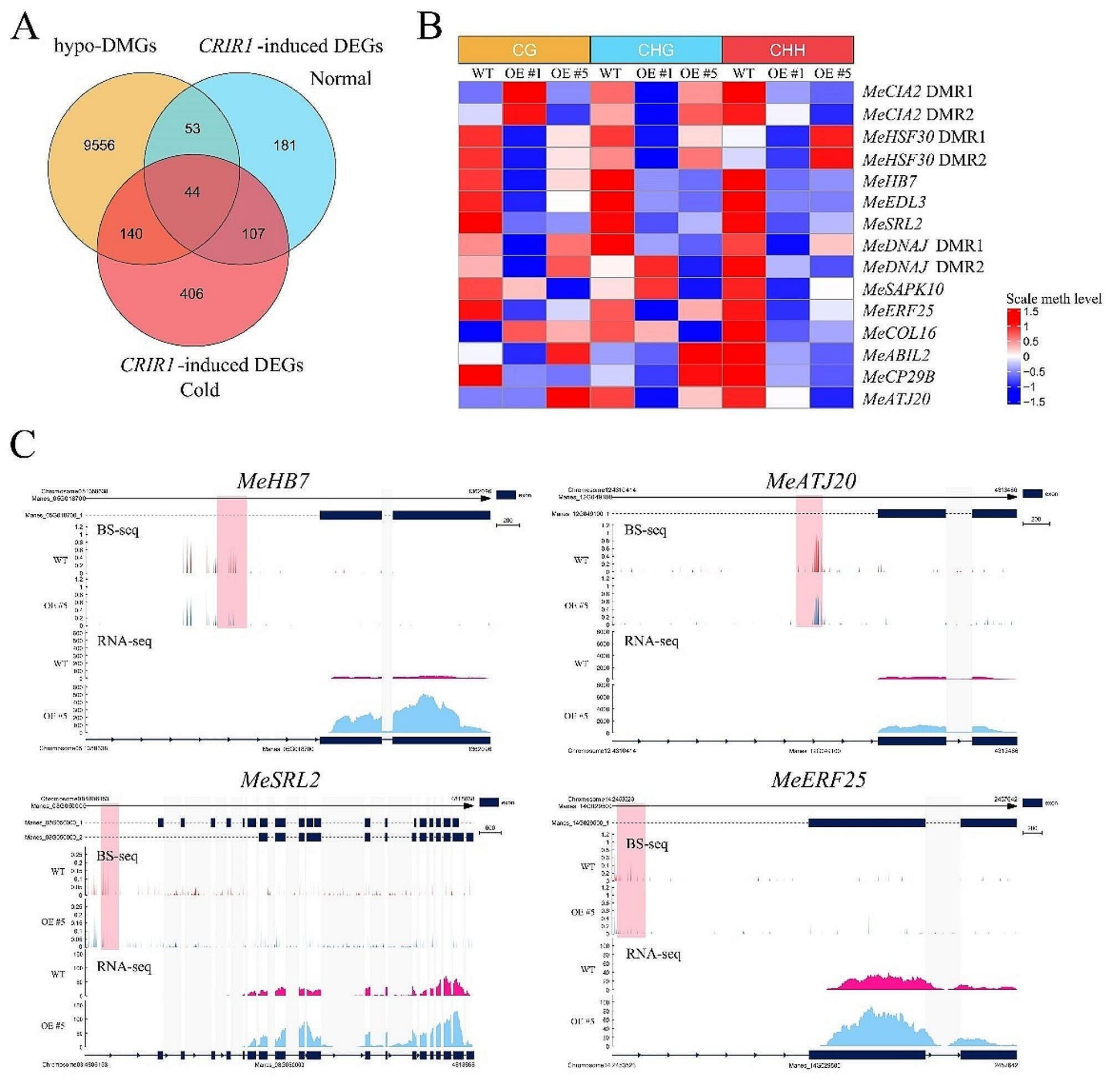
DEGs with altered DNA methylation in *CRIR1*-overexpressing plants. **(A)** Venn diagram showing overlap among hypo-DMGs and *CRIR1*-induced DEGs under normal and cold treatment conditions. **(B)** Heat map showing DNA methylation levels of *CRIR1*-induced hypo-DMGs in the CG, CHG, and CHH contexts of OE #1, OE #5 and WT plants. **(C)** mCHH methylation levels and expression levels of *MeHB7*, *MeATJ20*, *MeSRL2* and *MeERF25*. The regions with pink box represent DMRs

In order to investigate whether altered DNA methylation levels of *CRIR1*-overexpressing plants are related to changes in gene expression, an integrated analysis was performed on the transcriptome and epigenome data. In our previous study, RNA-sequencing was performed on WT and two OE lines cultivated under normal (N) and cold stress (C) conditions. A total of 385 and 697 *CRIR1*-induced DEGs were identified in OE plants grown under normal and cold conditions, respectively [25]. In this study, we mainly focused on analyzing the association between hypo-DMGs and *CRIR1*-induced DEGs. Venn diagram shows that hypo-DMGs and DEGs under different conditions highly overlap with each other, and a total of 44 hypo-DMGs are consistently hyperactivated by *CRIR1* under both normal and cold conditions (Fig. 4A). Among them, twelve genes encoding transcription factors, chaperone proteins, kinases, or RNA-binding proteins were selected for further analysis. As shown in Fig. 4B and C, *CRIR1*-overexpressing plants present decreased DNA methylation levels of *MeHB7*, *MeERF25*, *MeATJ20*, *MeSRL2*, *MeCP29B*, *MeSAPK10*, *MeABIL2* and *MeEDL3*, which are negatively correlated with gene transcription levels (Fig. S2). This indicates that hypo-methylation is an important factor of the transcription regulation of these genes in *CRIR1*-overexpressing plants. Among those genes simultaneously up-regulated, *MeSAPK10* encodes a Ser/Thr SnRK2 type kinase, and its homolog *OsSAPK10* in rice could directly interact with and phosphorylate *OsABF1*, *OsbZIP20* and *OsWRKY87*, and enhance their DNA-binding capacities with the promoters of downstream genes, thereby elevating the transcription of downstream genes and improving chilling, drought and salinity tolerance [31–33]. Belonging to an F-box protein family, EDL3 functions as a positive regulator in ABA-dependent signaling cascades that control anthocyanin accumulation under drought stress [34]. Over the past decade, significant progress has been made in the investigation of the role of ERF TF family in improving cold tolerance of plants [35]. Their expression levels in young leaves of OE and WT plants were confirmed by qRT-PCR (Fig. S2). *CRIR1* overexpressing in cassava leads to enhanced cold tolerance, facilitating the involvement of these genes in cold stress responses.

### *CRIR1*-induced genes are related to chlorophyll metabolism and photosynthesis

**Fig. 5:**
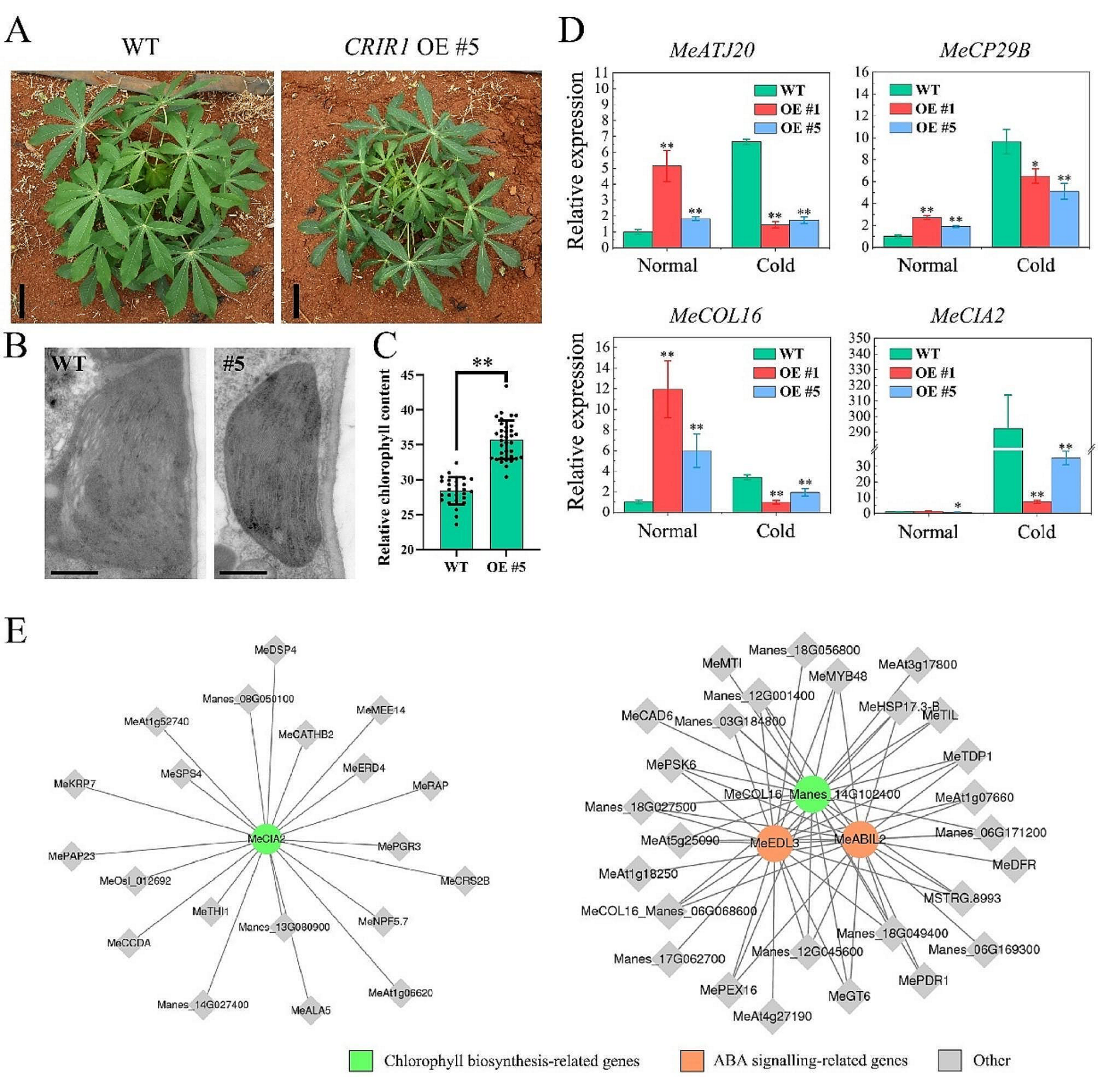
Expression and co-expression networks of chlorophyll metabolism and photosynthesis related genes. **(A)** Two-month-old *CRIR1* transgenic and WT plants grown in field. Bar = 10 cm. **(B)** Relative chlorophyll contents in the leaves of WT and OE #5 plants. SPAD values were recorded at three different positions on the same leaf, and the results are presented as mean values ± SD. **(C)** Transmission electron microscopic analysis of chloroplast membranes in WT and OE #5 young leaves under normal condition. Scale bar = 1 μm. **(D)** qRT-PCR examined the expression levels of the *MeATJ20*, *MeCP29B*, *MeCOL16* and *MeCIA2* genes in shoot tips under normal and cold treatment (4 °C, 48 h) conditions. Data are provided as means ± SD of three biological replicates, *P < 0.05, and **P < 0.01 by standard t-test. **(E)** Co-expression networks of genes obtained with WGCNA

We noticed that four genes relating to chlorophyll metabolism and photosynthesis, including *MeATJ20*, *MeCP29B*, *MeCOL16* and *MeCIA2*, are hypomethylated and upregulated in the *CRIR1*-overexpressing plants (Fig. 4B). *MeATJ20* encodes a chloroplast-targeted DnaJ proteins. Knockout of *ATJ20* in *Arabidopsis* will result in destabilized PSII complexes, decreased photosynthetic efficiency and loss control of balancing the redox reactions in chloroplasts [36]. The RNA-binding protein MeCP29B functions as a chloroplast ribonucleoprotein and plays a key role in the metabolism of chloroplast RNA [37]. CP29 protein in *Arabidopsis* is essential for cold stress tolerance with its capacity for stabilizing numerous chloroplast mRNAs [38]. *MeCOL16* is closely related to petunia *PhCOL16*, and its overexpression can lead to increased chlorophyll biosynthesis gene expression and higher chlorophyll levels in corollas of petunia [39]. MeCIA2 is a nuclear transcription factor with a C-terminal CCT motif, which can activate the expression of chloroplast transcription-, translation-, protein import-, and photosynthesis-related genes, thereby increasing the efficiency of protein import and synthesis in chloroplasts [40]. In *Arabidopsis*, *cia2* mutant was lighter green than wild-type, and showed reduced chlorophyll and carotenoid contents [41]. Interestingly, *CRIR1*-overexpressing plants are greener than WT plants, and exhibited higher levels of chlorophyll (Fig. 5A-B). Considering that the chloroplast size in the *cia2* mutant is slightly smaller than in wild-type of *Arabidopsis*, we examined the ultrastructure of mature leaves from WT and OE #5 via transmission electron microscopy. As shown in Fig. 5C, *CRIR1* overexpression line showed no phenotypic differences in chloroplast area compared with WT plant, but the chloroplast membranes density of OE plant was significantly higher than that of the WT plant. Further qRT-PCR assay showed that these four genes are hyperactivated in OE lines compared with WT, which is consistent with the results of transcriptome (Fig. 5D). Subsequently, a weighted gene co-expression network analysis (WGCNA) was performed to screen out *CRIR1* target genes related to chlorophyll metabolism. Finally, 20 genes were identified co-expression with *MeCIA2*. Among these genes, *NPF5*, *PGR3*, *RAP*, *MEE14*, *ERD4*, *SPS4*, *RAP23*, and *KRP7* are the most important hub genes involved in chlorophyll metabolism. In addition, the network diagram shows that *PSK6, MYB48*, *PEX16* and *GT6* are correlated with both chlorophyll metabolism-related genes, such as *MeCOL16*, and ABA signaling-related genes, such as *MeEDL3* and *MeABIL2* (Fig. 5E). All these data collectively provide a number of candidate genes with potential roles in the chlorophyll metabolism and photosynthesis control, and products of these genes are likely to mediate the *CRIR1*-induced cold tolerance in cassava.

## Discussion

Cassava is an essential calories source for more than 800 million people worldwide, making it important for food security and economic development. However, as a tropical and subtropical crop plant, cassava is highly sensitive to low temperatures and cannot survive long under freezing conditions. During the recent years, great advances have been made in the physiological and molecular investigations of cassava plants against cold stress [19]. Previously, we identified and characterized a lncRNA, named *CRIR1*, which function as a positive regulator of cassava plant responses to cold stress by inducing a range of stress-responsive genes [25]. However, it is still not clear that whether epigenetic regulation, like DNA methylation, is involved in *CRIR1*-induced gene expression in response to cold stress. In this study, we provided a comprehensive characterization of the DNA methylation changes in *CRIR1*-overexpressing plants and revealed a positive association between DNA methylation and *CRIR1*-mediated transcriptional activation downstream genes.

### Synergistic regulatory relationship between *CRIR1* and DNA methylation

DNA methylation is a conserved and important epigenetic mark involved in many biological processes [30]. During the last decade, it has been verified that lncRNAs can mediate DNA methylation among both mammals and plants [42, 43]. For example, lncRNA-*DACOR1* can recruit DNA methyltransferase and reprogram genome-wide DNA methylation in human colon cancer cells [44]. Similarly, it has been reported that lncRNA *ADAMTS9-AS2* can recruit DNMT1/3, inhibiting the proliferation and migration of esophageal cancer cells [45]. Human H19 lncRNA alters DNA methylation by binding to and inhibiting S-adenosylhomocysteine hydrolase (SAHH) [46]. In plants, RNA polymerase IV- and V-transcribed lncRNAs play a key role in the RNA-directed DNA methylation silencing pathway [47]. In *Arabidopsis*, lncRNA-*APOLO* can influence the expression of its neighboring gene PID by affecting chromatin loop formation and active DNA demethylation [48]. In this study, single-base resolution maps of DNA methylation of *CRIR1*-overexpressing plants were analyzed, with the finding that total DNA methylation levels of the *CRIR1*-overexpressing plants are similar to those levels of the wild-type plants. Interestingly, transgenic plant lines present very high numbers of hypomethylated DMRs. It is known that DNA demethylation, particularly in the promoter region, is often associated with locus-specific gene activation in plant development and stress responses [49]. This study shows that, among those 9,793 hypomethylated genes in *CRIR1*-overexpressing plants, 97 genes are upregulated under normal condition, 184 are upregulated after cold treatment, indicating that *CRIR1*-mediated DNA methylation dynamics and gene expression alteration are a complicated process with unknown mechanism. Condsidering that *CRIR1* does not alter the expression of methylase or demethylase genes, one possible mechanism is that *CRIR1* can directly or indirectly recruit DNA methyltransferases or demethylases to regulate the transcription of target genes.

### *CRIR1*-induced genes associated with chloroplast development and photosynthesis

Chloroplasts are indispensable organelles for photosynthesis in plants, providing the sites of chlorophyll synthesis and accumulation for photosynthetic products. In order to maintain normal photosynthesis, the development and functions of chloroplasts need to be well-regulated in the growth and stress responses of plants [50]. Many studies have shown that chilling can induce structural changes among chloroplasts, thus affecting their functions and development [50, 51]. Chloroplast organizations or photosynthesis-related genes were abundantly enriched in our WGCNA analysis results, such as *MeCP29B*, whose homolog *AtCP29A* can interact with and stabilize multiple chloroplast mRNAs that are important for limiting the effects of cold stress on chloroplast development [38]. *AtJ20*, which is a homolog of *MeATJ20* (a small chloroplast-targeted DnaJ protein) can not only participate in the stabilization of PSII supercomplexes but also be involved in ROS-induced stress responses of *Arabidopsis* [36]. Notably, increased mRNA levels of *MeCP29B* and *MeATJ20* genes in *CRIR1*-overexpressing plants were observed in this study, indicating that *MeCP29B* and *MeATJ20* genes can participate in *CRIR1*-mediated cold responses by stabilizing photosynthesis-related gene products at RNA and protein levels, respectively. It was also found that, two transcription factors among hypomethylated DMGs, *MeCIA2* and *MeCOL16*, are essential for the synthesis of chlorophyll [39, 40]. It is generally believed that chlorophyll plays an essential role in photosynthesis, directly limiting photosynthetic rates and crop yields. Our observations of dark-green phenotypes for the leaves of the *CRIR1*-overexpressing plant are mainly due to 25-50% higher level of chlorophyll relative to WT. We speculated that *CRIR1*-mediated cold tolerance may also be closely related to higher transcript levels of *MeCIA2* and *MeCOL16*, as well as their corresponding chlorophyll metabolism maintenance. However, the relationship between chlorophyll accumulation and cold tolerance in cassava is still unclear and this hypothesis remains to be further verified.

### Potential regulatory effects of *CRIR1* and transcription factors in cold stress responses

Transcription factors play a central role in regulating the expression of several stress-related genes, making them promising candidates in genetic engineering of crop plants [52]. In cassava, many TF families, including *MYB*, *WRKY*, *HD-ZIP*, *SPL* and *ERF/DREB*, are involved in abiotic stress responses, and many TF genes are associated with enhanced tolerance to cold and/or drought stresses [19, 23, 24, 53–55]. In this study, we found that the gene body of *MeHB7* and *MeERF25* was hypomethylated in *CRIR1*-overexpressing plants than in WT plants. Increased expression of these two genes correspond to decreased DNA methylation levels. *HD-ZIP* (*Homeo-Leucine Zipper*) genes, a unique class of TFs in higher plants, are involved in the regulation of plant development and stress responses [56]. Constitutively expressed *HD-ZIP* homologous transcription factors *HaHB1* from sunflower in *Arabidopsis*, confer cold tolerance via inducing several transcripts encoding glucanases and chitinases. Similarly, *AtHB13* from *Arabidopsis* can also promote the cold tolerance of plants by stabilizing cell membranes under freezing conditions [57]. AP2/ERF transcription factors constitute one of the largest gene superfamilies in plants and are involved in developmental processes of plants and their responses to multiple environmental stimuli [35]. For instance, it has been found that overexpression of *ERF40* genes in radish of transgenic *Arabidopsis* can alleviate oxidative damage of the plant under cold stress, thus increasing its cold tolerance [58]. 147 *ERF* members have been identified in cassava, and a number of *ERF* genes are involved in cold stress response [59]. However, whether *MeHB7* and *MeERF25* play a key role in *CRIR1*-mediated cold responses needs to be further verified.

## Materials and methods

### Plant materials and growth conditions

Cassava cultivar CV.60,444 (wild-type, WT) and *CRIR1*-overexpressing transgenic lines (#1 and #5) were used in this study. Uniform stem cuttings were cultivated in Murashige and Skoog (MS) medium containing 20 g/L sucrose (Fisher), 2 µmol/L CuSO_4_ and 3.0 g/L Gelrite in a growth room at 26℃ under a condition of 16 h light/8 h dark.

### Whole-genome bisulfite sequencing (WGBS) and methylation level analysis

Young leaves and shoot buds of 6-week-old *CRIR1*-overexpressing plants #1, #5 and WT plants (a total of three samples) were collected. The total DNA isolation, library preparation and bisulfite sequencing were performed by Guangzhou Genedenovo Biotechnology Co., Ltd (Guangzhou, China). Briefly, genomic DNAs were fragmented into 100-300 bp with Sonication (Covaris, Massachusetts, USA) and purified with MiniElute PCR Purification Kit (QIAGEN, MD, USA). Fragmented DNAs were then end repaired and a single “A” nucleotide was added to the 3’ end of each blunt fragment. Then these genomic fragments were ligated with methylated sequencing adapters. Fragments with adapters were bisulfite converted using the Methylation-Gold kit (ZYMO, CA, USA), with unmethylated cytosine converted to uracil through sodium bisulfite treatment. Finally, these converted DNA fragments were PCR amplified and sequenced using the Illumina HiSeqTM 2500. After removing the reads with adapters, undetermined reads, and low-quality reads, the obtained clean reads were mapped to the cassava reference genome using the default BSMAP software [60]. Then a custom Perl script was used to call methylated cytosines that were subsequently tested with a correction algorithm [61]. Methylated cytosine percentages in whole genome were calculated to determine the methylation level of each chromosome and different regions of the genome in each sequence context (CG, CHG and CHH). In order to assess different methylation patterns in different genomic regions, methylation profiles of flanking 2 kb regions and gene body were plotted based on the average methylation levels at each window. Bisulfite-seq data have been submitted to the National Center for Biotechnology Information (NCBI) with the accession number PRJNA736017.

### Differentially methylated region (DMR) analysis

In order to identify differentially methylated regions (DMRs) between samples, the minimum read coverage to call a methylation status for a base was set to 4. DMRs in each sequence context (CG, CHG and CHH) according to the following criteria: (1) For CG, GC number in each window ≥ 5, absolute value of methylation ratio difference ≥ 0.25, and q ≤ 0.05; (2) For CHG, GC number in each window ≥ 5, absolute value of methylation ratio difference ≥ 0.25, and q ≤ 0.05; (3) For CHH, GC number in each window ≥ 15, absolute value of methylation ratio difference ≥ 0.15, and q ≤ 0.05; (4) For all C, GC numbers in each window ≥ 20, absolute value of methylation ratio difference ≥ 0.2, and q ≤ 0.05.

### KEGG enrichment analysis of DMR-related genes

Kyoto encyclopedia of genes and genomes pathway enrichment analysis (http://www.kegg.jp/kegg/) was performed on DMR-related genes. An analysis result with p-value < 0.05 was considered statistically significant.

### Quantitative real-time PCR

After cold stress treatment (4℃, 24 h), young leaves and shoot buds of 4-week-old *CRIR1*-overexpressing plants and WT plants were harvested, and then frozen in liquid nitrogen for RNA extraction. Young leaves and shoot buds of non-stressed plants were collected as controls. Total RNAs were extracted using the RNAprep Pure Polysaccharide polyphenol Plant Total RNA Extraction Kit (Tiangen, Beijing, China), and first-strand complementary DNAs (cDNAs) were synthesized using the FastKing gDNA Dispelling RT SuperMix (Tiangen, Beijing, China). A quantitative real-time PCR was then performed with a Applied Biosystems StepOne™ and StepOnePlus™ Real-Time PCR Systems using the SYBR® Premix Ex Taq™ II (Tli RNaseH Plus) (Takara, Beijing, China). *MeACTIN* was used as a reference gene for normalizing transcript levels. All primers used in qPCR are listed in Table S1.

### Measurement of SPAD value of chlorophyll in cassava

To compare the relative chlorophyll content of *CRIR1*-overexpressing plants and WT plants, the SPAD-502 Plus (Konica Minolta Company, Japan) were used to measure the chlorophyll content of cassava leaves at 4 weeks. SPAD values were recorded at three different positions on the same leaf, and the results are presented as mean values.

### Electronic supplementary material

Below is the link to the electronic supplementary material.

Supplementary Material 1

Supplementary Material 2

Supplementary Material 3

## Data Availability

The datasets generated and analyzed during the current study are available in the NCBI repository with the primary accession code PRJNA736017, URL: https://www.ncbi.nlm.nih.gov/bioproject/PRJNA736017.

## References

[1] Zhang H, Lang Z, Zhu JK (2018). Dynamics and function of DNA methylation in plants. Nat Rev Mol Cell Biol.

[2] Lucibelli F, Valoroso MC, Aceto S (2022). Plant DNA methylation: an epigenetic mark in development, environmental interactions, and evolution. Int J Mol Sci.

[3] Gentry M, Hennig L (2014). Remodelling chromatin to shape development of plants. Exp Cell Res.

[4] Kankel MW, Ramsey DE, Stokes TL, Flowers SK, Haag JR, Jeddeloh JA, Riddle NC, Verbsky ML, Richards EJ (2003). Arabidopsis MET1 cytosine methyltransferase mutants. Genetics.

[5] Stroud H, Do T, Du J, Zhong X, Feng S, Johnson L, Patel DJ, Jacobsen SE (2014). Non-CG methylation patterns shape the epigenetic landscape in Arabidopsis. Nat Struct Mol Biol.

[6] Law JA, Jacobsen SE (2010). Establishing, maintaining and modifying DNA methylation patterns in plants and animals. Nat Rev Genet.

[7] Zhang X, Jacobsen SE (2006). Genetic analyses of DNA methyltransferases in *Arabidopsis thaliana*. Cold Spring Harb Symp Quant Biol.

[8] Gong Z, Morales-Ruiz T, Ariza RR, Roldán-Arjona T, David L, Zhu JK (2002). ROS1, a repressor of transcriptional gene silencing in Arabidopsis, encodes a DNA glycosylase/lyase. Cell.

[9] Ortega-Galisteo AP, Morales-Ruiz T, Ariza RR, Roldán-Arjona T (2008). Arabidopsis DEMETER-LIKE proteins DML2 and DML3 are required for appropriate distribution of DNA methylation marks. Plant Mol Biol.

[10] Kidokoro S, Shinozaki K, Yamaguchi-Shinozaki K (2022). Transcriptional regulatory network of plant cold-stress responses. Trends Plant Sci.

[11] Maruyama K, Todaka D, Mizoi J, Yoshida T, Kidokoro S, Matsukura S, Takasaki H, Sakurai T, Yamamoto YY, Yoshiwara K, Kojima M, Sakakibara H, Shinozaki K, Yamaguchi-Shinozaki K (2012). Identification of cis-acting promoter elements in cold- and dehydration-induced transcriptional pathways in Arabidopsis, rice, and soybean. DNA Res.

[12] Jia Y, Ding Y, Shi Y, Zhang X, Gong Z, Yang S (2016). The cbfs triple mutants reveal the essential functions of CBFs in cold acclimation and allow the definition of CBF regulons in Arabidopsis. New Phytol.

[13] Olate E, Jiménez-Gómez JM, Holuigue L, Salinas J (2018). NPR1 mediates a novel regulatory pathway in cold acclimation by interacting with HSFA1 factors. Nat Plants.

[14] Egbune EO, Ezedom T, Orororo OC, Egbune OU, Avwioroko OJ, Aganbi E, Anigboro AA, Tonukari NJ (2023). Solid-state fermentation of cassava (*Manihot esculenta* Crantz): a review. World J Microbiol Biotechnol.

[15] Sonnewald U, Fernie AR, Gruissem W, Schläpfer P, Anjanappa RB, Chang SH, Ludewig F, Rascher U, Muller O, van Doorn AM, Rabbi IY, Zierer W (2020). The Cassava Source-Sink project: opportunities and challenges for crop improvement by metabolic engineering. Plant J.

[16] Lin ZJD, Taylor NJ, Bart R (2019). Engineering disease-resistant cassava. Cold Spring Harb Perspect Biol.

[17] Mohidin S, Moshawih S, Hermansyah A, Asmuni MI, Shafqat N, Ming LC (2023). Cassava (Manihot esculenta Crantz): a systematic review for the pharmacological activities, traditional uses, nutritional values, and Phytochemistry. J Evid Based Integr Med.

[18] El-Sharkawy MA (2004). Cassava biology and physiology. Plant Mol Biol.

[19] Li S, Zhao P, Yu X, Liao W, Peng M, Ruan M (2022). Cell signaling during drought and/or cold stress in cassava. Trop Plants.

[20] An D, Yang J, Zhang P (2012). Transcriptome profiling of low temperature-treated cassava apical shoots showed dynamic responses of tropical plant to cold stress. BMC Genomics.

[21] An F, Li G, Li QX, Li K, Carvalho LJ, Ou W, Chen S (2016). The comparatively proteomic analysis in response to cold stress in cassava plantlets. Plant Mol Biol Rep.

[22] An D, Ma Q, Yan W, Zhou W, Liu G, Zhang P (2016). Divergent regulation of CBF regulon on cold tolerance and plant phenotype in cassava overexpressing Arabidopsis CBF3 gene. Front Plant Sci.

[23] An D, Ma Q, Wang H, Yang J, Zhou W, Zhang PJP. Cassava C-repeat binding factor 1 gene responds to low temperature and enhances cold tolerance when overexpressed in Arabidopsis and cassava. 2017;94:109–24. 10.1007/s11103-017-0596-6 28258553

[24] Ruan MB, Guo X, Wang B, Yang YL, Li WQ, Yu XL, Zhang P, Peng M (2017). Genome-wide characterization and expression analysis enables identification of abiotic stress-responsive MYB transcription factors in cassava (*Manihot esculenta*). J Exp Bot.

[25] Li S, Cheng Z, Dong S, Li Z, Zou L, Zhao P, Guo X, Bao Y, Wang W, Peng M (2022). Global identification of full-length cassava lncRNAs unveils the role of cold-responsive intergenic lncRNA 1 in cold stress response. Plant Cell Environ.

[26] Wang H, Beyene G, Zhai J, Feng S, Fahlgren N, Taylor NJ, Bart R, Carrington JC, Jacobsen SE, Ausin I (2015). CG gene body DNA methylation changes and evolution of duplicated genes in cassava. Proc Natl Acad Sci U S A.

[27] Xiao L, Lu L, Zeng W, Shang X, Cao S, Yan H (2022). DNA methylome and LncRNAome analysis provide insights into mechanisms of genome-dosage effects in autotetraploid cassava. Front Plant Sci.

[28] Shim S, Lee HG, Park OS, Shin H, Lee K, Lee H, Huh JH, Seo PJ (2022). Dynamic changes in DNA methylation occur in TE regions and affect cell proliferation during leaf-to-callus transition in Arabidopsis. Epigenetics.

[29] Zhu W, Yang C, Liu Q, Peng M, Li Q, Wang H, Chen X, Zhang B, Feng P, Chen T, Zeng D, Zhao Y (2023). Integrated analysis of DNA methylome and transcriptome reveals epigenetic regulation of cold tolerance in *litopenaeus vannamei*. Int J Mol Sci.

[30] Huang H, Liu R, Niu Q, Tang K, Zhang B, Zhang H, Chen K, Zhu JK, Lang Z (2019). Global increase in DNA methylation during orange fruit development and ripening. Proc Natl Acad Sci U S A.

[31] Yan L, Baoxiang W, Jingfang L, Zhiguang S, Ming C, Yungao X, Bo X, Bo Y, Jian L, Jinbo L, Tingmu C, Zhaowei F, Baiguan L, Dayong X, Bello BK (2021). A novel SAPK10-WRKY87-ABF1 biological pathway synergistically enhance abiotic stress tolerance in transgenic rice (*Oryza sativa*). Plant Physiol Biochem.

[32] Shu Y, Zhang W, Tang L, Li Z, Liu X, Liu X, Liu W, Li G, Ying J, Huang J, Tong X, Hu H, Zhang J, Wang Y (2023). ABF1 positively regulates rice chilling tolerance via inducing trehalose biosynthesis. Int J Mol Sci.

[33] Baoxiang W, Bo X, Yan L, Jingfang L, Zhiguang S, Ming C, Yungao X, Bo Y, Jian L, Jinbo L, Tingmu C, Zhaowei F, Baiguan L, Dayong X, Bello BK (2022). A novel mechanisms of the signaling cascade associated with the SAPK10-bZIP20-NHX1 synergistic interaction to enhance tolerance of plant to abiotic stress in rice (*Oryza sativa* L). Plant Sci.

[34] Koops P, Pelser S, Ignatz M, Klose C, Marrocco-Selden K, Kretsch T (2011). EDL3 is an F-box protein involved in the regulation of abscisic acid signalling in *Arabidopsis thaliana*. J Exp Bot.

[35] Ritonga FN, Ngatia JN, Wang Y, Khoso MA, Farooq U, Chen S (2021). AP2/ERF, an important cold stress-related transcription factor family in plants: a review. Physiol Mol Biol Plants.

[36] Chen KM, Holmström M, Raksajit W, Suorsa M, Piippo M, Aro EM (2010). Small chloroplast targeted DnaJ proteins are involved in optimization of photosynthetic reactions in *Arabidopsis thaliana*. BMC Plant Biol.

[37] Uberegui E, Hall M, Lorenzo Ó, Schröder WP, Balsera M (2015). An Arabidopsis soluble chloroplast proteomic analysis reveals the participation of the executer pathway in response to increased light conditions. J Exp Bot.

[38] Kupsch C, Ruwe H, Gusewski S, Tillich M, Small I, Schmitz-Linneweber C (2012). Arabidopsis chloroplast RNA binding proteins CP31A and CP29A associate with large transcript pools and confer cold stress tolerance by influencing multiple chloroplast RNA processing steps. Plant Cell.

[39] Ohmiya A, Oda-Yamamizo C, Kishimoto S (2019). Overexpression of CONSTANS-like 16 enhances chlorophyll accumulation in petunia corollas. Plant Sci.

[40] Yang CY, Yan WY, Chang HY, Sun CW (2022). Arabidopsis CIA2 and CIL have distinct and overlapping functions in regulating chloroplast and flower development. Plant Direct.

[41] Sun CW, Huang YC, Chang HY (2009). CIA2 coordinately up-regulates protein import and synthesis in leaf chloroplasts. Plant Physiol.

[42] Huang W, Li H, Yu Q, Xiao W, Wang DO (2022). LncRNA-mediated DNA methylation: an emerging mechanism in cancer and beyond. J Exp Clin Cancer Res.

[43] Urquiaga MCO, Thiebaut F, Hemerly AS, Ferreira PCG (2020). From trash to luxury: the potential role of plant LncRNA in DNA methylation during abiotic stress. Front Plant Sci.

[44] Somasundaram S, Forrest ME, Moinova H, Cohen A, Varadan V, LaFramboise T, Markowitz S, Khalil AM (2018). The DNMT1-associated lincRNA DACOR1 reprograms genome-wide DNA methylation in colon cancer. Clin Epigenetics.

[45] Liu D, Wu K, Yang Y, Zhu D, Zhang C, Zhao S (2020). Long noncoding RNA ADAMTS9-AS2 suppresses the progression of esophageal cancer by mediating CDH3 promoter methylation. Mol Carcinog.

[46] Zhou J, Yang L, Zhong T, Mueller M, Men Y, Zhang N, Xie J, Giang K, Chung H, Sun X, Lu L, Carmichael GG, Taylor HS, Huang Y (2015). H19 lncRNA alters DNA methylation genome wide by regulating S-adenosylhomocysteine hydrolase. Nat Commun.

[47] Wierzbicki AT, Haag JR, Pikaard CS (2008). Noncoding transcription by RNA polymerase Pol IVb/Pol V mediates transcriptional silencing of overlapping and adjacent genes. Cell.

[48] Ariel F, Jegu T, Latrasse D, Romero-Barrios N, Christ A, Benhamed M, Crespi M (2014). Noncoding transcription by alternative RNA polymerases dynamically regulates an auxin-driven chromatin loop. Mol Cell.

[49] Li Y, Kumar S, Qian W (2018). Active DNA demethylation: mechanism and role in plant development. Plant Cell Rep.

[50] Li JY, Yang C, Tian YY, Liu JX (2022). Regulation of chloroplast development and function at adverse temperatures in plants. Plant Cell Physiol.

[51] Song Y, Feng L, Alyafei MAM, Jaleel A, Ren M (2021). Function of chloroplasts in plant stress responses. Int J Mol Sci.

[52] Baillo EH, Kimotho RN, Zhang Z, Xu P (2019). Transcription factors associated with abiotic and biotic stress tolerance and their potential for crops improvement. Genes (Basel).

[53] Wei Y, Liu W, Hu W, Yan Y, Shi H (2020). The chaperone MeHSP90 recruits MeWRKY20 and MeCatalase1 to regulate drought stress resistance in cassava. New Phytol.

[54] Yu X, Guo X, Zhao P, Li S, Zou L, Li W, Xu Z, Peng M, Ruan M (2023). A homeodomain-leucine zipper I transcription factor, MeHDZ14, regulates internode elongation and leaf rolling in cassava (*Manihot esculenta* Crantz). Crop J.

[55] Li S, Cheng Z, Li Z, Dong S, Yu X, Zhao P, Liao W, Yu X, Peng M (2022). MeSPL9 attenuates drought resistance by regulating JA signaling and protectant metabolite contents in cassava. Theor Appl Genet.

[56] Gong S, Ding Y, Hu S, Ding L, Chen Z, Zhu C (2019). The role of HD-Zip class I transcription factors in plant response to abiotic stresses. Physiol Plant.

[57] Cabello JV, Arce AL, Chan RL (2012). The homologous HD-Zip I transcription factors HaHB1 and AtHB13 confer cold tolerance via the induction of pathogenesis-related and glucanase proteins. Plant J.

[58] Li C, Mao B, Wang K, Xu L, Fan L, Wang Y, Li Y, Ma Y, Wang L, Liu L (2023). RsERF40 contributes to cold stress tolerance and cell expansion of taproot in radish (*Raphanus sativus* L). Hortic Res.

[59] Fan W, Hai M, Guo Y, Ding Z, Tie W, Ding X, Yan Y, Wei Y, Liu Y, Wu C, Shi H, Li K, Hu W (2016). The ERF transcription factor family in cassava: genome-wide characterization and expression analyses against drought stress. Sci Rep.

[60] Xi Y, Li W (2009). BSMAP: whole genome bisulfite sequence MAPping program. BMC Bioinformatics.

[61] Lister R, Pelizzola M, Dowen RH, Hawkins RD, Hon G, Tonti-Filippini J, Nery JR, Lee L, Ye Z, Ngo QM, Edsall L, Antosiewicz-Bourget J, Stewart R, Ruotti V, Millar AH, Thomson JA, Ren B, Ecker JR. Human DNA methylomes at base resolution show widespread epigenomic differences. Nature. 2009;462(7271):315–22. 10.1038/nature08514 PMC2857523 19829295

