# Supplementary material for: Integrated analysis of DNA methylome and transcriptome revealing epigenetic regulation of CRIR1-promoted cold tolerance
Source: BMC Plant Biol. 2024 Jul 5;24:631. doi: 10.1186/s12870-024-05285-0 (PMC11225538; doi:10.1186/s12870-024-05285-0)
Supplement: Supplementary file 3 — Supplementary Material 3 [file 12870_2024_5285_MOESM3_ESM.docx]

**Supplementary Information**

**Integrated analysis of DNA methylome and transcriptome revealing epigenetic regulation of *CRIR1*-promoted cold tolerance**

Zhibo Li^1^, Wenjuan Wang^1,2^, Xiaoling Yu^1^, Pingjuan Zhao^1^, Wenbin Li^1^, Xiuchun Zhang^1^, Ming Peng^1^, Shuxia Li^1^*, Mengbin Ruan^1^*

*^1^National Key Laboratory for Tropical Crop Breeding, Key Laboratory of Biology and Genetic Resources of Tropical Crops, Institute of Tropical Bioscience and Biotechnology, Sanya Research Institute of Chinese Academy of Tropical Agricultural Sciences, Haikou, 571101, P.R.China*

*^2^College of Tropical Crops, Hainan University, Haikou, 570228, P.R.China*

**Corresponding authors*: Mengbin Ruan, ruanmengbin@itbb.org.cn; Shuxia Li, lishuxia@itbb.org.cn


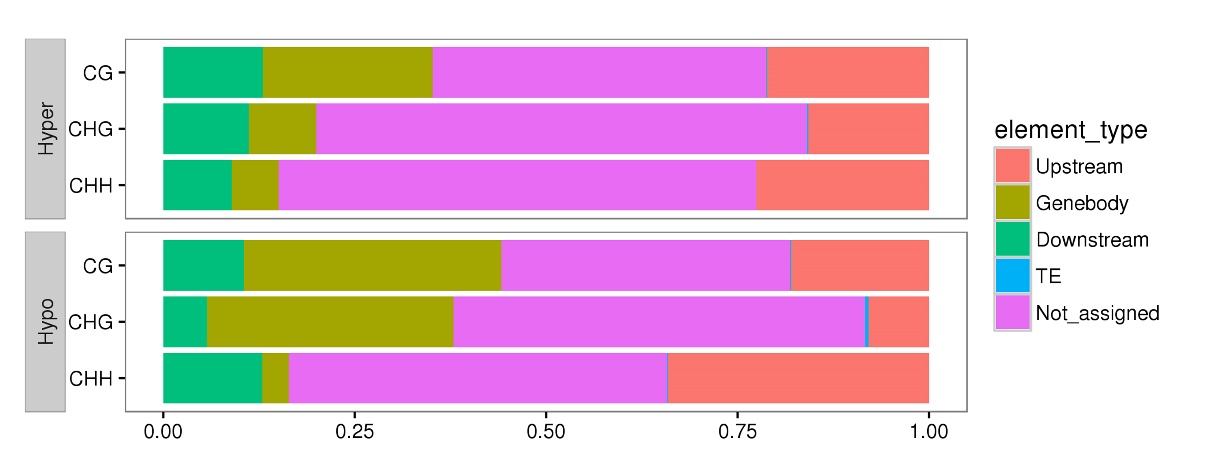


**Fig.S1** DMR distribution in genic and transposable element (TE) regions. Relative fractions of DMRs to total DMRs are presented.


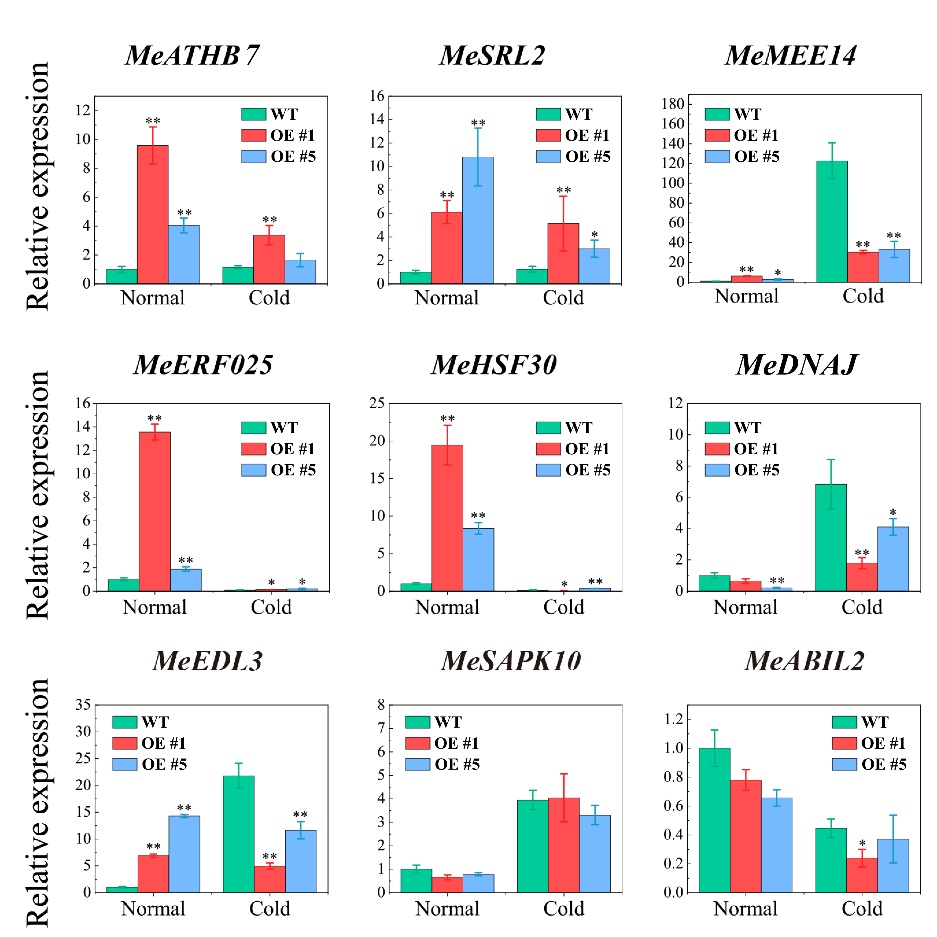


**Fig.S2** Relative expression levels of selected genes in WT and OE plants. Data are provided as means [±](https://www.so.com/s?q=%C2%B1&psid=de1ba8369b35ed96c0ae33496c985230&eci=&nlpv=zzzc_base_1&src=pdr_guide_3.5&ls=n114c1aa49a) SD of three biological replicates, *P<0.05, and **P<0.01 by standard t-test.
